# Reprogramming aging: genetically enhanced mesenchymal progenitor cells show systemic rejuvenation in primates

**DOI:** 10.1093/lifemedi/lnaf022

**Published:** 2025-06-19

**Authors:** Hongkun Wang, Yuanyuan Du

**Affiliations:** Hangzhou Institute of Medicine (HIM), Chinese Academy of Sciences, Hangzhou 310000, China; Hangzhou Institute of Medicine (HIM), Chinese Academy of Sciences, Hangzhou 310000, China

Aging is a systemic, multifactorial process that manifests as tissue degeneration, reduced stem-cell competence, chronic inflammation, and a progressive decline in physiological function. Although cell therapy has emerged as a promising avenue to counter age-related decline [1, 2], achieving sustained rejuvenation in primate models has remained elusive. In a study published in *Cell*, Lei et al. demonstrate that senescence-resistant mesenchymal progenitor cells (SRCs), engineered via Forkhead box O3 (FOXO3) modification, confer geroprotective effects in aged monkeys [3]. This work presents a new perspective for stem-cell-based rejuvenation research and suggests a potential strategy for aging interventions.

FOXO3 is a well-established regulator of longevity, stress resistance, and stem-cell maintenance [4–6]. In a pioneering effort to reprogram aging-related genetic circuits, Liu’s group introduced two phospho-null mutations (S253A and S315A) into the FOXO3 locus, generating engineered human embryonic stem cells that, upon mesenchymal differentiation, gave rise to progenitor cells with enhanced stress resilience and self-renewal capacity—designated as senescence-resistant cells (SRCs). These cells exhibited enhanced proliferative potential, reduced secretion of senescence-associated secretory phenotype factors, and increased heterochromatin stability, all without evidence of transformation or tumorigenicity.

Administering SRCs intravenously to aged cynomolgus monkeys over a 44-week period led to a cascade of restorative changes. Compared to wild-type mesenchymal cells, SRCs more effectively reversed age-related changes across the brain, immune system, bone, skin, and reproductive tissues. Multi-modal assessments—behavioral, histological, transcriptomic, and methylomic—consistently indicated biological age reversal.

Notably, SRC-treated monkeys exhibited improved cognitive function, restored cortical architecture, and enhanced hippocampal connectivity. Bone density increased, periodontal degeneration was mitigated, and immune cell transcriptional profiles shifted toward a youthful state. At the molecular level, transcriptomic aging clocks showed an average reversal of 3.34 years with SRCs, while DNA methylation clocks corroborated these effects in multiple tissues. Furthermore, the authors observed the restoration of reproductive system health. In both male and female monkeys, SRC treatment reduced senescent markers, enhanced germ cell preservation, and reversed transcriptional aging clock across ovaries and testes. Single-cell transcriptomics revealed that oocytes, granulosa cells, and testicular germ cells responded particularly well, rejuvenating by up to 5–6 years. These findings offer new insights into addressing reproductive aging and fertility decline.

**Figure 1.**
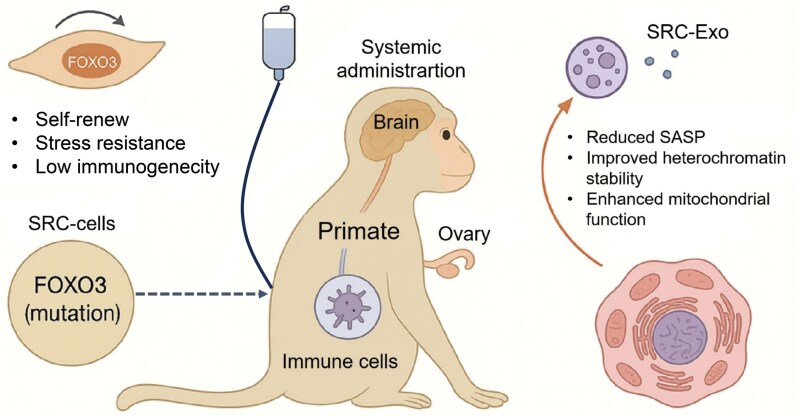
FOXO3-enhanced mesenchymal progenitor cells rejuvenate primate tissues via systemic and exosomal pathways.

The benefits of SRCs extended beyond direct cell integration. Their secreted extracellular vesicles, or exosomes (SRC-Exo), potentially carried enriched cargoes including geroprotective proteins and metabolites such as spermine [7]. SRC-Exo delivered in mice delayed aging across liver, lung, kidney, and skeletal muscle by 2–4 months and reduced histological markers of senescence and inflammation (Fig. 1) . In human cell models, SRC-Exo reversed senescence phenotypes in neurons, hepatocytes, and ovarian stromal cells, suggesting that much of the systemic rejuvenation may be mediated via paracrine signaling.

This study illustrates an allogeneic stem-cell platform with observed multi-organ rejuvenative capacity. Importantly, it leverages FOXO3—an evolutionarily conserved longevity factor—rather than synthetic overexpression systems, enabling stable and safety-compliant gene editing. The authors’ application of multi-omic aging clocks introduces a valuable quantitative aspect, contributing to a framework for assessing future interventions. The findings support the idea that aging may be reversible at the systems level, suggesting that cellular products could potentially serve as next-generation gerotherapeutics.

Several important questions remain to be explored in future studies. A critical next step is to conduct long-term tracking to determine the durability of the rejuvenative effects conferred by SRC infusion. While SRC-derived exosomes appear to play an important role in mediating these benefits, it remains to be clarified whether other mechanisms—such as direct immune modulation or metabolic rewiring—also contribute to the systemic effects observed. Moreover, it is essential to determine which age groups or physiological states (e.g. younger adults or peri-menopausal individuals) respond most favorably to SRC therapy. Taken together, the work by Lei et al. explores a novel framework for stem cell-based geroprotection and offers a valuable reference for future studies in anti-aging interventions through cell transplantation.
